# Hereditary Multiple Intestinal Atresia: A Case Report and Review of the Literature

**DOI:** 10.7759/cureus.30870

**Published:** 2022-10-30

**Authors:** Maher M Al-Zaiem, Rawan S Alsamli, Ethar A Alsulami, Ruba F Mohammed, Mohammad I Almatrafi

**Affiliations:** 1 Department of Pediatric Surgery, Maternity and Children Hospital, Makkah, SAU; 2 College of Medicine and Surgery, Umm Al-Qura University, Makkah, SAU

**Keywords:** silastic stent, multiple anastomosis, low birth weight, preterm, hereditary multiple intestinal atresia

## Abstract

Hereditary multiple intestinal atresia (HMIA) is a rare form of intestinal atresia that affects the gastrointestinal tract from the pylorus to the rectum. A few cases have been reported in the literature. Here, we report the case of a three-day-old girl who was referred to our hospital as a case of upper intestinal obstruction. After initial resuscitation, the radiological examination revealed pyloric obstruction, multiple intra-abdominal calcifications, and rectal atresia making the diagnosis of HMIA most likely. Exploratory laparotomy revealed multiple intestinal atresia, the first started at the pylorus, the second was at the level of the duodenojejunal junction, and there were also multiple small bowel atresias. The colon was a cord-like structure, and there was rectal atresia. Multiple resections of the atretic intestinal segment followed by multiple anastomoses, terminal ileostomy, and the use of a trans-anastomotic tube were performed. In this study, the clinical picture, radiological findings, and management are described and compared to the findings reported in the literature.

## Introduction

One-third of all cases of neonatal intestinal obstruction are caused by intestinal atresia. Multiple intestinal atresia is a rare form of intestinal atresia characterized by the presence of several atretic segments in the small or large bowel, resulting in signs of intestinal obstruction in infants, such as vomiting, abdominal bloating, and failing to pass meconium. Moreover, multiple atresia is associated with an increased risk of mortality [1].

Hereditary multiple intestinal atresia (HMIA), a severe congenital disorder, was first defined by Guttman and colleagues in 1973 [2]. The gene known to cause HMIA is the *tetratricopeptide repeat domain-containing protein 7A* (*TTC7A*) gene [3]. Mutation in *TTC7A *is often associated with severe intestinal defects and severe combined immunodeficiency (SCID) and inflammatory bowel disease [4]. Multiple intestinal atresia in newborns is usually treated with multiple anastomoses. Subsequently, total parenteral nutrition is needed over the long term or permanently [4-6].

To date, most studies have been reported in Europe and the United States, with limited cases reported in Saudi Arabia. Thus, the rarity and treatment challenges of the disease led us to present a case and review the literature.

## Case presentation

A preterm three-day-old girl was born by cesarean section to healthy non-consanguineous parents at 36 weeks of gestation; her birth weight was 1.7 kg. She was referred to our hospital as a case of intestinal obstruction from a peripheral hospital where an oral contrast study was done, and it was diagnosed as duodenal obstruction. However, the prenatal follow-up did not raise the diagnosis of intestinal obstruction.

**Figure 1:**
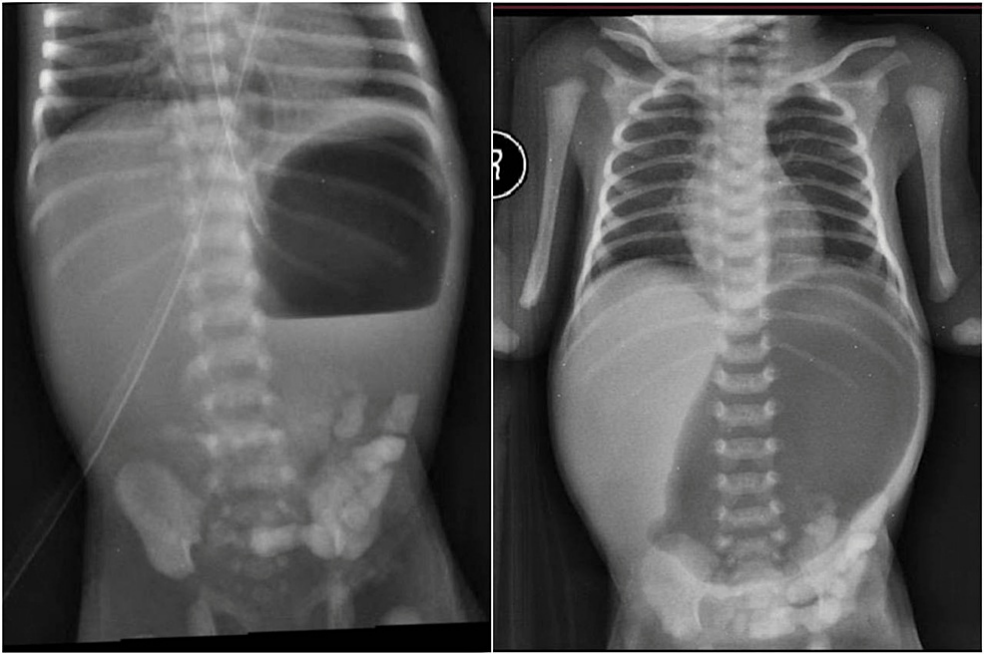
Plain X-ray showed a huge, dilated stomach.

**Figure 2:**
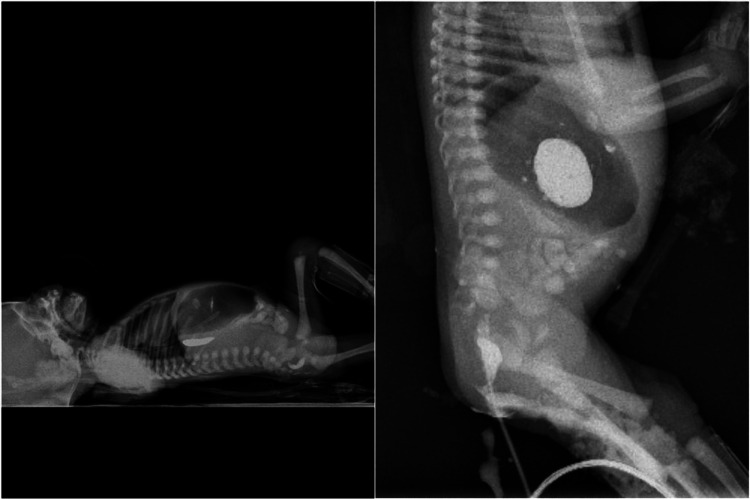
Retained contrast in the stomach and intestinal luminal calcification. A contrast enema showing rectal obstruction.

Upon arrival, the baby presented with vomiting and failure to pass meconium. On examination, the patient had stable respiratory and hemodynamic status with no evident dysmorphic features. The abdomen was soft, lax, and not distended. A rectal tube was inserted but failed to pass beyond 3 cm. Laboratory studies were unremarkable. A plain abdominal X-ray showed a hugely dilated stomach (Figure 1) with remnants of contrast material from the previous hospital seen in the stomach, and there were multiple intra-abdominal calcifications, which seemed to be intraluminal (Figure 2). Contrast material was injected through the anus but arrested at 3 cm from the anal verge indicating rectal atresia.

**Figure 3:**
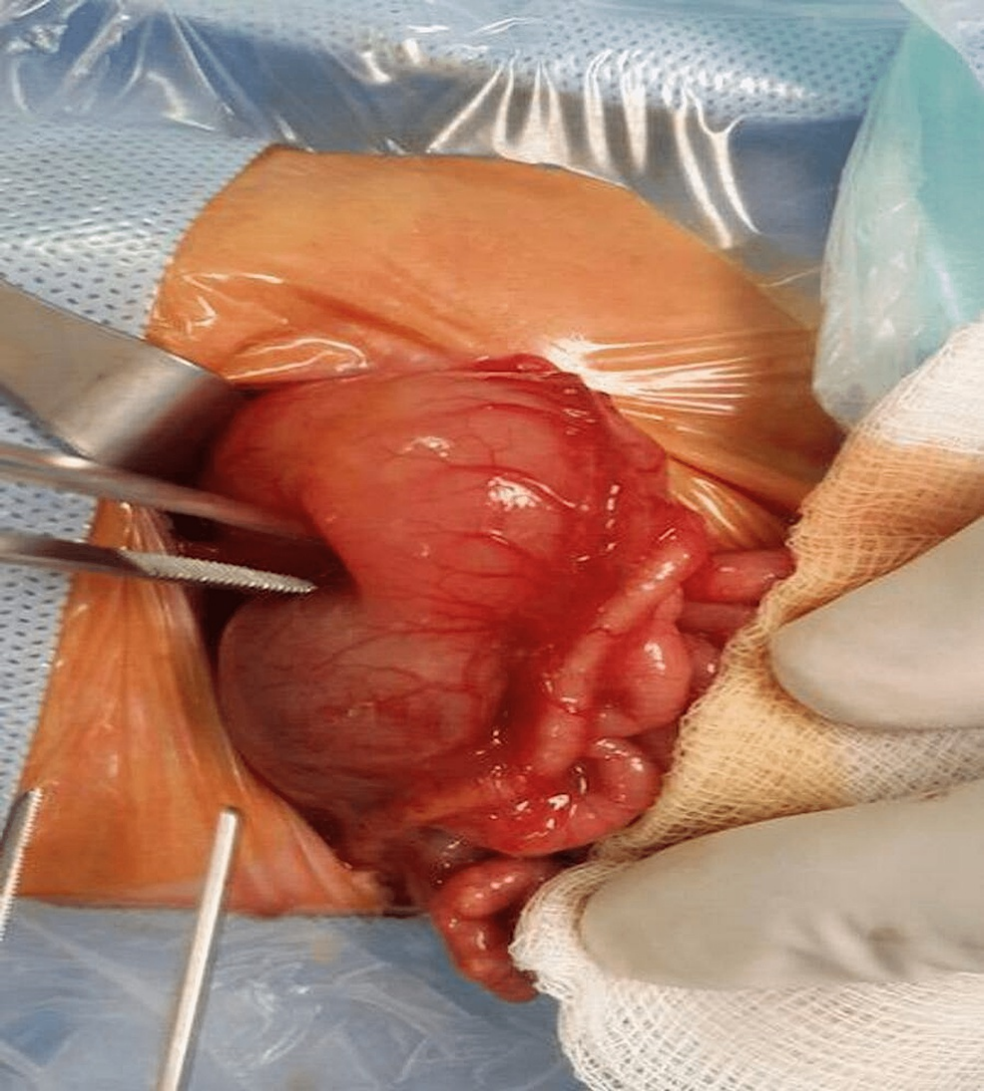
Intraoperative image showing the pyloric obstruction, cord stricture colon, and calcified material in the small bowel.

After a period of stabilization, she underwent an exploratory laparotomy, which revealed multiple intestinal atresia, the first atresia was at the pylorus, and the second atresia was at the duodenojejunal junction. There was also multiple atresia of the small bowel, with multiple completely atretic cord-like segments of the intestine. The remaining part of the small intestinal was segmented, with every segment measuring about 2-3 cm containing whitish calcified material. The colon was also found to be completely atretic, with a closed lumen like a cord (Figure 3). Multiple resections of the small atretic areas were performed, keeping only the segments of the intestine longer than 4 cm, followed by 10 intestinal anastomoses. At the level of the pylorus, pyloroplasty was performed to bypass the atretic segment, while a stricturoplasty was done at the level of the duodenojejunal junction. A silastic tube was passed through all the anastomoses as a stent, the proximal part of the tube was in the duodenum, and the distal part was exteriorized out through a terminal jejunostomy. The closed colonic cord was left as is.

Postoperatively, the baby was shifted to the neonatal intestinal care unit and was kept on total parenteral nutrition with intravenous antibiotics. On follow-up, there was a minute whitish secretion through the trans-anastomotic tube. Multiple trials of feeding the baby through the nasogastric tube failed. Subsequently, the condition of the baby deteriorated due to gram-negative sepsis in the blood, and, unfortunately, she died after three weeks of the surgery. In our case, the short life of the baby did not allow further investigations to confirm the presence of SCID.

## Discussion

The rarest form of multiple atresia is HMIA which affects the gastrointestinal tract from the stomach to the rectum [4,7]. The exact etiology and pathogenesis of HMIA are not completely understood [4]. Based on the pathological finding, the suggested cause of HMIA is the malformation of the gastrointestinal tract, rather than the presence of ischemic changes, which is usually associated with mesenteric defects caused by vascular insults [4,8]. The most likely explanation for the disease is autosomal recessive transmission, as suggested by Bilodeau et al. [4] and Ishii et al. [9].

Prenatal diagnosis is still an issue. Ultrasound can raise suspicion of HMIA, especially in the presence of polyhydramnios, gastric dilatation, and intraluminal calcification; however, it cannot distinguish HMIA from other types of intestinal atresia despite advancements in the detection of intestinal anomalies [4,10,11]. However, prenatal follow-up was missed in our case. HMIA is usually confirmed intraoperatively. However, in our case, the diagnosis of HMIA was made preoperatively due to the presence of combined radiological findings: the excessive dilatation of the stomach with no distal aeration indicating pyloric obstruction, the presence of intraluminal calcification on the plain X-ray, and the conformation of rectal atresia by contrast enema. The presence of these combined signs is considered pathognomonic of HMIA.

The aim of the surgical intervention should be to restore the continuity of the gastrointestinal tract and maintain the maximum length of the viable bowel [4,7,8,12]. In our case, this was achieved by resections of the very small obstructed areas, preserving multiple small areas of the intestine, followed by multiple primary anastomoses. The use of trans-anastomotic tubes to stent multiple anastomoses, as in our case, has also been reported in the literature [7,8,12]. Unfortunately, all reported cases in the literature died due to intestinal failure and sepsis which is related to low immunity [4,12].

**Table 1:** A summary of previously reported cases.

Author	Ours	Case 1 [13]			Case 2 [14]	Case 3 [15]	Case 4 [16]					Case 5 [17]	Case 6 [18]	Case 7 [4]		Case 8 [19]	Case 9 [20]	Case 10 [21]	Case 11 [20]	Case 12 [21]	
Number of cases	1	Three siblings in one family			1	1	Five patients in two families					2	1	2		1	4	1	1	2	
Gestational age	36	37	37	38	36	NA	23	NA	NA	NA	38	30	38	36	38	35	NA	Term	37	35	36
Polyhydramnios	NA	Positive	NA	Positive	NA	NA	Present	NA	NA	NA	Present	Present	Present	Present	Present	NA	NA	NA	Present	Present	Present
Antenatal diagnosis	NA	NA	Suspected	Dilated small bowel	NA	NA	Present	NA	NA	NA	Present	Present	Distended, echogenic bowel	Distended, calcified bowel	NA	NA	NA	NA	Intestinal obstruction	Polyhydramnios, abdominal cyst	Duodenal atresia, abdominal mass
Immunodeficiency	NA	Present	Present	Present	Present	Present	Fanconi anemia	NA	NA	Not proven	Present	Present	Present	Present	Present	Present	Present	Present	present	Present	Present
Outcome	Died at week 3	Died at week 8	Died at week 15	Died at 7 months	Died at 2 months	Died at 8 months	Died at 16 years	Died	Terminated pregnancy at 23	Died	NA	NA due to discharge	Died at 2 months	Died at 42	Died at 64	Lives	Died	Died	Died at 13 months	Died at 6 months	Died at 3 months

The comparison in Table 1 is carried out according to gestational age, presence of polyhydramnios, antenatal diagnosis, immunodeficiency, and outcomes [22]. Our patient passed away three weeks after receiving supportive care, followed by surgical intervention. HMIA is often associated with SCID according to Moreno who first prescribed this condition which contributes to the death of these babies [13]. So far, there is no reported survival among previously reported cases. Further, there is no curative treatment. Hence, even with adequate medical and surgical intervention, it is deemed a lethal condition. As the most probable cause of HMIA is an autosomal recessive mode of inheritance, the absence of near consanguinity does not rule out autosomal recessive transmission [23].

Medical supportive management and early surgical intervention to restore the continuity of the gastrointestinal tract through multiple resections and anastomoses is the usual course of treatment based on the literature and our own experience. Moreover, a small bowel transplant or bone marrow transplant or even gene therapy may be a viable therapy option; however, further research is required to confirm this line of treatment. Although this is currently a rare disease, each case study can provide us the opportunity to learn more about the true pathophysiology.

## Conclusions

HMIA should be considered in cases of neonatal intestinal obstruction with radiological signs combined with pyloric obstruction, rectal obstruction, and multiple intraluminal intestinal calcifications. The family should be aware of the prognosis. Further immunological and genetic workup in the future might clarify the disease and improve the prognosis.
